# Modeling a microbial community and biodiversity assay with OBO Foundry ontologies: the interoperability gains of a modular approach

**DOI:** 10.1093/database/bau132

**Published:** 2015-01-28

**Authors:** Philippe Rocca-Serra, Ramona Walls, Jacob Parnell, Rachel Gallery, Jie Zheng, Susanna-Assunta Sansone, Alejandra Gonzalez-Beltran

**Affiliations:** ^1^Oxford e-Research Centre, University of Oxford, OX1 3QG, Oxford, UK, ^2^The iPlant Collaborative, University of Arizona, Thomas J. Keating Bioresearch Building, 1657 East Helen St., Tucson, AZ 85721, USA, ^3^NEON, Inc. 1685 38th St., Ste. 100, Boulder, CO 80301, USA, ^4^University of Arizona, 1311 E. 4th St. Tucson, AZ 85721, USA and ^5^Department of Genetics and Institute of Biomedical Informatics, Perelman School of Medicine, University of Pennsylvania, Philadelphia, PA 19104, USA

## Abstract

The advent of affordable sequencing technology provides for a new generation of explorers who probe the world’s microbial diversity. Projects such as Tara Oceans, Moorea Biocode Project and Gut Microbiome rely on sequencing technologies to probe community diversity. Either targeted gene surveys (also known as community surveys) or complete metagenomes are evaluated. The former, being the less costly of the two methods, relies on the identification of specific genomic regions, which can be used as a proxy to estimate genetic distance between related species in a Phylum. For instance, 16 S ribosomal RNA gene surveys are used to probe bacterial communities while internal transcribed spacer surveys, for example, can be used for probing fungal communities. With the explosion of projects and frenzy to explore new domains of life, scientists in the field have issued guidelines to report minimal information (following a checklist), ensuring that information is contextualized in a meaningful way. Yet the semantics of a checklist are not explicit. We demonstrate here how a tabular template can be used to collect information on microbial diversity using an explicit representation in the Resource Description Framework that is consistent with community agreed-upon knowledge representation patterns found in the Ontology for Biomedical Investigations.

## Introduction

The times when natural scientists could only rely on an acute sense of observation, pencil and paper to document biological diversity never seemed so distant, A new golden age of exploration of the Earth’s biodiversity is powered by the latest generation of sequencing instruments and sequence analysis algorithms. Ten years after Craig Venter’s Sargasso Sea expedition, projects such as the Earth Microbiome Project (1), Tara Oceans (http://oceans.taraexpeditions.org), the Moorea Biocode project (http://mooreabiocode.org) and the American Gut (http://americangut.org) are gathering information at unforgiving rates.

Two main sequencing procedures are currently used to characterize environmental samples. The first approach (known as ‘environmental gene survey’ or ‘targeted, amplicon based survey’) relies on the knowledge of specific genomic sequences known to harbor variable regions that can be used to assess species richness in a given sample. The other approach, more onerous per sample, consists of performing a deep sequencing of all genomic DNA isolated from an environmental sample; this is known as ‘metagenome sequencing’.

For the first technique, a number of genes are now routinely used to explore the diversity of a particular domain of microbial life. People with an interest in bacterial population will focus on the 16 S ribosomal RNA gene and its subregions (for instance V5-V6 hypervariable region). The internal transcribed spacer gene is used in particular for investigating Fungal life forms, while the mitochondrial cytochrome oxidase I (COI) gene is mostly used to characterize animals. Whichever gene is selected, the principle is the same: polymerase chain reaction (PCR) primers are designed to amplify the whole gene or a subregion within it. The amplicon is then used as input to a sequencing library preparation process.

To foster proper data preservation and maximize data reuse, annotation requirement guidelines have been issued under the Genomic Standards Consortium (GSC) initiative. The checklist, known as Minimum Information for any (x) Sample (MIxS) (2), itemizes elements to report about samples, sampling conditions and preservation, sample processing and processes such as data acquisition and processing. Depending on the nature of the organism, the environment, the community or the assay used, the guidelines define specific requirements. Furthermore, specific database handles for each of the annotation requirements have been defined by the International Nucleotide Sequence Database Collaboration, a consortium bringing together the major institutional sequence data archives across the world. Therefore, Sequence Read Archive (SRA) files, Genbank (3) records but also Investigation Study Assay format (ISA-Tab) documents (4) may contain a MIxS compliant metadata payload relying on those elements. Although these shared annotation guidelines ensure a similar level of annotation, the diversity of formats and interfaces somewhat limits data integration capabilities.

We describe here how resources of the Open Biological and Biomedical Ontologies (OBO) Foundry (5) have been used to provide a semantic framework enabling the presentation of biodiversity information as linked data. The approach offers means to ease meshing data from dissimilar origin but of similar scope, which could be accessed through the linked open data cloud. Furthermore, we outline how gains can be made by taking advantage of the different but complementary and specialized ontology modules available under the OBO Foundry.

## Materials and Methods

Domain experts and ontology developers reviewed: (i) the MIxS checklists, (ii) the soil microbial data collection and metadata tracking worksheet (http://goo.gl/nE9zPk) from the National Ecological Observatory Network (NEON) (http://www.neoninc.org), (iii) the ISA-Tab archive representing a targeted gene survey of gut microflora in children of Africa and Italy (a dataset identified by BII-S-7) published by de Filippo *et al*. (6), available from http://goo.gl/USjDDW, alongside the relevant ISA configurations (a set of xml documents defining annotation requirements and workflow organization using ISA syntax, used by ISAtools to create table and validate input), and explored their semantic representations for mapping to the following OBO Foundry resources: the Ontology for Biomedical Investigations (OBI) (7) and the Population and Community Ontology (PCO) (8).

Knowledge elicitation was carried out using CmapTools (http://cmap.ihmc.us/) as means to rapidly prototype representations based on key entities common to all OBO Foundry resources. This meant deconstructing the data capture table and bin field headers into elements such as process, material, quality and data item.

**Figure 1.**
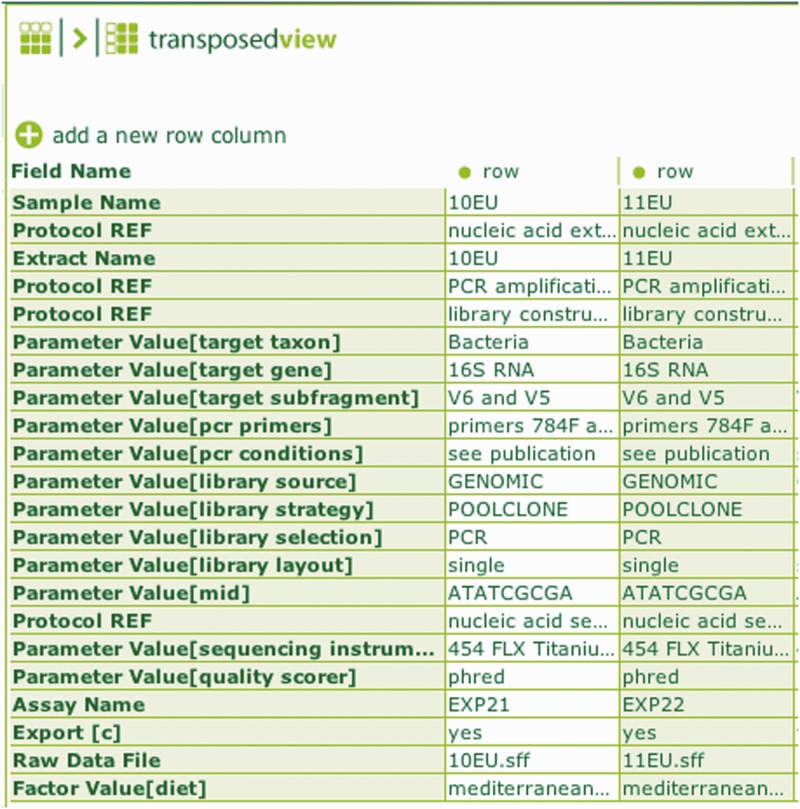
A screenshot of ISAcreator showing the transposed assay table indicating the overall sample processing workflow and relevant MiXs labels

In the case of BII-S-7, an ISA-Tab representation of a 16S rRNA barcoding assay has been used to deposit data to NCBI SRA. The mapping work was greatly facilitated by the fact that an ISA configuration table had been defined already for ‘environmental gene survey’ using the ‘nucleotide sequencing’ assay definition. In that specific configuration, MIxS tags specific to the assay had been mapped into a workflow indicated by the ordered sequence of ISA syntactic elements. Furthermore, taking advantage of the ISAconverter, the experience of mapping into an existing schema, such SRA xml schema, proved to be of great help in informing the definition of classes under the Basic Formal Ontology (BFO) framework and facilitated the processing and characterization of the NEON data collection template (Fig. 1).

For both use cases, the purpose of this work was to clarify the semantics of the data collection tables, focusing on making explicit the meaning of each variable’s relative positions and possible dependencies as well as directionality of relations among columns.

## Results

**Figure 2.**
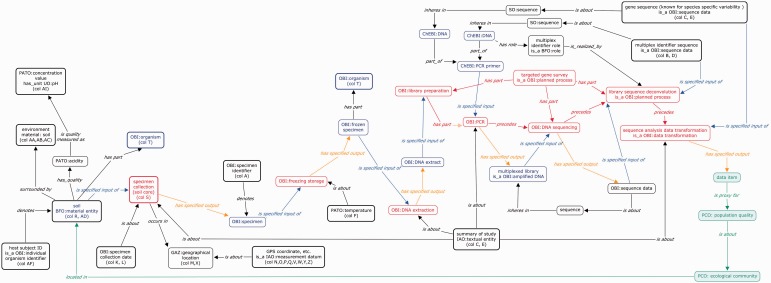
A graphical overview representing the various steps of an environmental gene survey, from the sampling step down to the data analysis

The conceptual map generated and presented in Figure 2 summarizes one of the main outcomes of these mapping efforts into a BFO based semantic representation framework.

The basic pattern for this representation is one defined by the OBI group, where an assay is defined as having a specified input of type Material, a specified output of type Data and achieves a specific objective. In this instance, a basic objective of this assay is to produce sequence data, so it is legitimate to rely on ‘OBI_020008’ (‘sequence analysis objective’ term) to satisfy the assay ‘achieves planned objective’ axiom. However, because the true goal is to provide information that can be used to gauge the diversity of the microbial population located in a given environmental sample, following OBI ontology design pattern for assays requires the declaration of a specific objective, such as ‘biodiversity assessment objective’. The representation simply builds on this motif based on the mapping of the MIxS guidelines in the ISA-Tab framework when defining the specific ISA configuration. As ISA table defines an experimental workflow process as a set of nodes (Materials or Data), the leftmost node is a material node and the rightmost node is a data node, in line with OBI assay declaration specifying inputs and outputs. ISA nodes are interspersed by ‘Protocol REF’ elements that represent processes whose types are annotated with OBI terms. The order in which they appear in the ISA assay table reflects the actual order of execution. In the OWL representation, the ‘precedes’ or ‘follows’ relations can be relied on to do this.

-  ‘targeted gene survey’
      achieves_objective some ‘biodiversity assessment objective’
      has part some ‘library preparation’
      has_part some ‘DNA sequencing’
      has_part some (‘library sequence deconvolution’
          and precedes some (‘sequence analysis data transformation’ and has_specified_output some
      (‘data item’ and is about ‘population quality’)))
-  ‘library preparation’
      has part some ‘polymerase chain reaction’
      has_specified_input some ‘DNA extract’
      has_specified_input some ‘multiplexing sequence identifier’
      precedes ‘DNA sequencing’
      has_specified_output’ some ‘single fragment library’
-  ‘polymerase chain reaction’
      has_specified_input some ‘forward pcr primer’
      has_specified_input some ‘reverse pcr primer’
      realizes o concretizes some ‘PCR program’

The mapping revealed gaps in coverage in several of the supporting ontologies (indicated in italics). For instance, multiplex identifier, target gene, target subfragment are missing from the representations from OBI. Also, neither OBI nor any OBO Foundry resources contain the necessary classes to represent accurately the type of data resulting from such an assay. The second outcome of the work is therefore the identification of coverage gaps in OBI and PCO, prompting the creation of several new term requests whose processing is underway by the respective ontologies.

Finally, Resource Description Framework (RDF) conversion of the tables have been performed using linkedISA converter (https://github.com/ISA-tools/linkedISA) for the BII-S-7 archive in order to further validate the benefits on building a semantic version of the data set. Although this initial conversion relies on the default settings, a mapping extension to support the representation shown in the concept map has been devised. It is currently functional but requires the new classes be officially released (see Table 1) in the respective ontologies in order for the resulting linked data files to be completed.

**Table 1.** Current term requests logged in the respective term tracker (OBI terms are available since OBI release-2014-12-03, subversion revision 3955).

Class label	Class definition	Target ontology
targeted gene survey	is an assay which aims to provide information about taxonomic information and community diversity by mean of sequencing specimen genomic regions used as marker of identity or diversity	OBI
multiplexing	a planned process which consists in running a set of samples as a pool in one single instrument run of a process while retaining the ability to associate individual results to each of the samples	OBI
library sequence deconvolution	is a data transformation which uses sequence alignment and ‘multiplex identifier sequence’ information to pull together all reads belonging to a given single sample following the sequencing of a multiplexed library which combining several samples in one sequencing event	OBI
PCR program	is a plan specification which is executed during a PCR by a thermal cycler instrument that will iterate through the changes in temperature and duration of each of the annealing, denaturation, elongation steps	OBI
multiplex identifier sequence	is a nucleic acid sequence which is used in a ligation step of library preparation process to allow pooling of samples while maintaining ability to identify individual source material	OBI
OTU matrix	OTU matrix is a data item, organized as a table, where organismal taxonomic units, computed by sequence analysis and genetic distance calculation, are counted in a set of biological or environmental samples. The table is used to appraise biodiversity of a population or community of living organism	PCO
target gene	is a data item about a coding genomic region which is the focus of a planned process such as an assay.	OBI
target subfragment	is a data item about a genomic region which is the focus of a planned process such as an assay.	OBI

## Discussion

This study case demonstrates the benefits of modular approaches and reuse of existing ontology design patterns. Reusing the assay design pattern devised by OBI, the knowledge elicitation with environmental science domain experts led to two outcomes. First, the validation of a tabular template for data collection and second, the creation of an explicit semantic representation of those templates, extending the OBI assay design pattern with specific elements from the domain, namely sequencing techniques applied to biodiversity studies. The experience gained by OBI in modeling assay and data production is directly applicable and benefits a new community of users. Re-using a motif in a new domain does not preclude it from being specific enough to include a sufficient level of information. In fact, the work described here results in complete disambiguation of the notion of ‘barcoding’ as met in the field of sequencing application and biodiversity studies, thus resolving a polymorphic and ambiguous use of the terminology. Leaving aside the true meaning of barcode identifier, a set of bars which could be scanned by an optical reading at checkout tills for product identification, while building on the analogy of use, the notion of ‘barcode’ in life science applications covers distinct meanings to depict two very distinct uses, as detailed next. We focus first on the more mundane use where ‘barcoding’ is synonymous to ‘multiplexing’. In the context of next generation sequencing multiplexing is a process by which several samples are sequenced as a pool in one single sequencing run. Sequences can then be deconvoluted thanks to a unique sequence tag (the barcode) associated with a specific sample used during a ligation step of the library preparation (9). Fragments of the genomic DNA from a given sample are ligated to a unique short nucleic acid sequence. Since all reads derived from that template will begin with this unique sequence of nucleotides, sequence alignment algorithms can easily pull all reads with this signature from a complex mixture. The technique has been developed to optimize sequencer occupancy and throughput, thus reducing costs. Although ‘multiplexing’ samples in a library is widely used, it does not mean it is an essential part of the sequencing assay. Although this technique proved to be extremely effective, its catchy denomination as ‘barcode’ is a source of confusion. The GSC and MIxS standards currently recommend abandoning ‘barcode’ as a term to address this issue and promote the more accurate ‘multiplex identifier’ designation, which encapsulates the true function of the sequence tag while removing any ambiguity caused by the other meaning of ‘barcoding’. ‘DNA barcoding’, in contrast, is a molecular biology technique used to help in taxonomic identification (https://www.ebi.ac.uk/ena/about/sra_format_1_5). The technique’s underlying principle (which has caused some controversy), is to rely on known genes as a proxy for species identification or biodiversity assessment, using sequence variations as indirect measurement of interspecies distance or species richness in a given sample. Hence, genes such as COI are used to characterized Eukaryota (10), while other genes are widely used for other organisms (e.g. 16 S ribosomal RNA for Bacteria). The technique relies on the specific amplification of genomic regions by PCR for analysis. Hence, the designation of ‘targeted gene survey’ is considered more accurate to describe the procedure. From a global viewpoint, we have shown that assays from very distinct domains can be represented using one single motif, making interrogation of data simple when moving from one domain to the next. According to the OBI assay design pattern, assays generate data sets or data items that are about some entity. In the case of biodiversity studies, an assay such as a targeted gene survey will generate sequence data, whose analysis will provide insights about a community of organisms living in a particular environment. The central element of the modeling is the need for the assay representation to link back to the underlying biology and the reasons for performing the assay in the first place. The purpose of the assay is not to produce sequence data as such. Rather, it is to produce an ‘information content entity’ that is about a community of living organism and that denotes some quality of that assembly of organisms in a given environment. More specifically, the information content entity in question could be an Operational Taxonomic Unit Matrix (OTU matrix) resulting from sequence analysis software such as Mothur (11) or QIIME (12). That matrix can be used as a proxy for a quality of the population under study, typically its ‘diversity’. Hence, the necessity to liaise and coordinate with resources such as PCO for locating the suitable material entities and qualities for use in the assay definition and, when necessary, request the creation of such classes. From a representation standpoint, it is essential for OBI and other resources which describe assays, that they do not stop simply at the raw output of the assay, but rather strive to capture the central aspects a data item provides about the biological entity of interest to the assay. The representation therefore distinguishes between the library preparation steps from the sequencing step and the sequence data analysis steps. It is also important to consider the semantics of the different conversions offered here. The ISA SRA converter for this type of assay strived to map entities for their most suitable objects. Yet, as the SRA schema (https://www.ebi.ac.uk/ena/about/sra_format_1_5) does not allow for granular annotation at the level of the SRA library protocol, annotations related to library creation such as PCR primers and conditions are lumped into the textual description of the object, making retrieval more difficult. As comparison, the SRA xml generated by ENA webin tool (13) places the information as ‘sample attributes’. Although this allows retrieval, the semantics is confusing and leaves room for interpretation. Is a PCR primer a property of Sample or more accurately, an input to library preparation process?

This matters as several libraries, one per targeted genomic region, are often derived from a single sample to monitor biodiversity at different phylum level, and difference in parsing can result in ambiguities about sample sizes and sample identity.

The conversion to RDF/OWL following the ISA template discussed here forces an exact, unambiguous mapping, which is of better value in the long run for preservation of such records. We can therefore consider the following queries:

What assays can be used to study biodiversity?
    select?a where {?a achieves_objective ‘biodiversity assessment objective’}

Which PCR primers can be used to study biodiversity?
    select?x,?y where {
    ?x ‘is_specified_input’ ‘targeted gene survey’.
    ?y ‘realizes o concretizes ‘forward pcr primer’ or ‘reverse pcr primer’
}

Finally, the work typifies the kind of synergistic development that can occur from relying on foundational resources and established patterns. Those resources can provide significant time gains but above all, consistency in representations, which in turn ensure consistency in query and can facilitate data discovery. The methodology and cross domain interaction showcase the portability of the approach, the efficiency of the methodologies in place, from term request to term triage and dispatch. The present work also outlines entire new areas of modeling, which will require attention and for which coordination between interested parties will be needed in order to deliver optimal gains. For instance, sequence data analysis methods specific to biodiversity studies as well as key metrics, indexes and graphs are currently missing, still leaving important gaps in the semantic network. As stated before, BFO-based resources such as OBI or PCO offer patterns and can be used as mid-level ontologies for developing suitable extensions. Alternately, both resources can be the recipient of term requests in order to extend the breadth and depth of the semantic support currently offered by those efforts.
